# Robust GABAergic Regulation of the GnRH Neuron Distal Dendron

**DOI:** 10.1210/endocr/bqac194

**Published:** 2022-12-02

**Authors:** Xinhuai Liu, Robert Porteous, Allan E Herbison

**Affiliations:** Centre for Neuroendocrinology and Department of Physiology, University of Otago School of Biomedical Sciences, Dunedin 9054, New Zealand; Centre for Neuroendocrinology and Department of Physiology, University of Otago School of Biomedical Sciences, Dunedin 9054, New Zealand; Centre for Neuroendocrinology and Department of Physiology, University of Otago School of Biomedical Sciences, Dunedin 9054, New Zealand; Department of Physiology, Development and Neuroscience, University of Cambridge, Cambridge CB2 3EG, UK

**Keywords:** GnRH, GABA receptor, GCaMP6, dendrite

## Abstract

The amino acid transmitter γ-aminobutyric acid (GABA) is suspected to play an important role in regulating the activity of the gonadotropin-releasing hormone (GnRH) neurons controlling fertility. Rodent GnRH neurons have a novel dendritic compartment termed the “*distal dendron*” through which action potentials pass to the axon terminals and where inputs from the kisspeptin pulse generator drive pulsatile GnRH secretion. Combining *Gnrh1-*Cre mice with the Cre-dependent calcium sensor GCaMP6 and confocal imaging of acute brain slices, we examined whether GABA regulated intracellular calcium concentrations ([Ca^2+^]) in the GnRH neuron distal dendron. Short puffs of GABA on the dendron evoked either a monophasic sustained suppression of [Ca^2+^] or a biphasic acute elevation in [Ca^2+^] followed by the sustained suppression. Application of muscimol to the dendron replicated the acute elevation in [Ca^2+^] while baclofen generated the sustained suppression. Robust GABA_B_ receptor-mediated inhibition was observed in 80% to 100% of dendrons recorded from females across the estrous cycle and from approximately 70% of dendrons in males. In contrast, the GABA_A_ receptor–mediated excitation was rare in males and varied across the estrous cycle, being most prominent at proestrus. The activation of GABA_B_ receptors potently suppressed the stimulatory effect of kisspeptin on the dendron. These observations demonstrate that the great majority of GnRH neuron distal dendrons are regulated by GABAergic inputs in a sex- and estrous cycle–dependent manner, with robust GABA_B_ receptor-mediated inhibition being the primary mode of signaling. This provides a new, kisspeptin-independent, pathway for the regulation of pulsatile and surge modes of GnRH secretion in the rodent.

Recent studies have highlighted the importance of the gonadotropin-releasing hormone (GnRH) neuron dendron in the control of gonadotropin secretion in rodents (1). This structure undertakes 2 important functions: First, it carries action potentials responsible for the GnRH surge propagating from the proximal dendrites of GnRH neurons to their axon terminals in the median eminence. Second, the distal aspect of the dendron, lying beneath the arcuate nucleus, receives an episodic input from the kisspeptin neuron pulse generator to drive the pulsatile release of GnRH. Thus, the distal dendron is a key compartment of the GnRH neuron and appears to represent a locus at which both surge and pulsatile LH secretion can be controlled (2). Although kisspeptin regulation of the dendron occurs through volume transmission (3), substantial direct synaptic innervation exists at the distal dendron (4). Indeed the highest density of synaptic inputs to a GnRH neuron is found at the distal dendron (2). However, very little is known about the neural regulation of the distal dendron, with a recent study finding that it was not modulated by glutamate, neurokinin B, or dynorphin (3).

The role of γ-aminobutyric acid (GABA) in the regulation of GnRH secretion has been a focus of investigation for more than 4 decades. Studies have reported substantial effects both of GABA_A_ and GABA_B_ receptor modulation on GnRH and luteinizing hormone (LH) secretion in multiple species as well as on GnRH neuron activity in rodents (5–12). However, without knowledge of the existence of the GnRH neuron dendron, this large body of work has been interpreted almost exclusively in terms of GABAergic modulation of the GnRH neuron cell body.

In the present study, we sought to examine whether GABAergic inputs may also be active at the newly identified GnRH neuron distal dendron. The small size of the dendron makes it extremely difficult to measure its electrical activity with conventional approaches. However, using the measurement of intracellular calcium as a proxy for excitability, it is possible to combine the Cre-dependent calcium sensor GCaMP6 with GnRH-Cre mice and in vitro confocal imaging to record the activity of the dendron directly (3, 13). We report here that mouse dendron activity is robustly modulated by GABA in an estrous cycle- and sex-dependent manner.

## Materials and Methods

### Animals and Stereotaxic Injection of Adeno-associated Virus (AAV9-GCaMP6)

Male and female C57BL/6 *Gnrh1-Cre* mice (14) aged 152 ± 15 days were used for experiments. All mice were provided with environmental enrichment under conditions of controlled temperature (22 ± 2 °C) and lighting (12-hour light/12-hour dark cycle; lights on at 06:00 hours and off at 18:00 hours) with ad libitum access to food (Teklad Global 18% Protein Rodent Diet 2918, Envigo) and water. Daily vaginal cytology over a period of at least 2 weeks was used to monitor the estrous cycle stage. All animal experimental protocols were approved by the University of Otago, New Zealand (96/2017).

The *Gnrh1-Cre* mice were given stereotaxic injections of Cre-dependent GCaMP6s into the preoptic area as described previously (13). In brief, mice were anesthetized with 2% isoflurane, given local lidocaine (4 mg/kg, subcutaneously [s.c.]) and carprofen (5 mg/kg, s.c.) and placed in a stereotaxic apparatus in which 1.5 µL of AAV9.CAG.Flex.GCaMP6s.WPRE.SV40 (1.7 × 10^13^ GC/mL; Penn Vector Core) was injected into the rostral preoptic area (AP 0.1, ML 0, D 0.43) over the course of 10 minutes. The injection needle was left in place for 10 minutes before and after injecting the virus. Carprofen (5 mg/kg body weight, s.c.) was administered for postoperative pain relief. Seven days after recovery from stereotaxic surgery, animals were group-housed and used for brain slice experiments 4 to 5 weeks later.

### Brain Slice Preparation and Confocal Imaging

Mice were killed by cervical dislocation, decapitated, and the brain quickly removed with the optic tract being peeled off. The dorsal surface of the brain was then glued to a vibratome cutting stage (VT1200s, Leica) and submerged in ice-cold (< 2 °C), high-sucrose, artificial cerebrospinal fluid (aCSF) cutting solution with an osmolarity of 320 mOsmol and the following components (in mM): 75 NaCl, 75 sucrose, 2.5 KCl, 20 HEPES, 15 NaHCO_3_, 0.25 CaCl_2_, 6 MgCl_2_, and 25 D-glucose, bubbled with 95% O_2_/5% CO_2_. The vibratome blade was positioned to just touch the median eminence of the hypothalamus and then a single 500-µm-thick para-horizontal slice prepared. The brain slice was incubated for approximately 12 minutes in sucrose-containing cutting solution at 34 ± 1 °C and then transferred into normal aCSF maintained at 27 ± 1 °C and incubated for at least 1 hour before being transferred into a recording chamber. The aCSF contained (in mM): 118 NaCl, 3 KCl, 10 HEPES, 25 NaHCO_3_, 2.5 CaCl_2_, 1.2 MgCl_2_, and 11 D-glucose, bubbled with 95% O_2_/5% CO_2_.

The brain slice was positioned ventral surface uppermost within the recording chamber and held between 2 meshes to allow both surfaces of the brain slice to be continuously perfused with oxygenated aCSF. All experiments were performed at 27 ± 1 °C (Inline Solution Heater, SH-27B, Warner Instrument) with a perfusion flow rate of approximately 1.5 mL/minute (Peristaltic Pump, Gilson Miniplus3, John Morris Scientific Ltd). Imaging was performed with an Olympus FV1000 confocal microscope fitted with a 40×, 0.8 NA objective lens and 3× zoom. GCaMP6 was excited with a 488 nm Argon laser (Melles Griot). Emitted light was detected by a photomultiplier tube after passing through a bandpass filter (505-605 nm). The confocal aperture was wide open during Ca^2+^ imaging experiments to collect maximum emitted fluorescence.

Test compounds were dissolved in aCSF and locally puff-applied with a patch pipette (4-6 MΩ) at low pressure (∼1 psi) controlled by a Pneumatic Picopump (PV821, World Precision Instruments) for 10 to 90 seconds. The tip of the puff pipette was positioned 30 to 100 µm above the surface of the slice and on the right side of the observation field of 40× objective, where GCaMP6-containing GnRH neuronal processes were imaged. To determine whether GnRH neuron dendrons were able to exhibit evoked Ca^2+^ alterations, 20 mM KCl-containing aCSF (300 mOsmol) was locally puff-applied for 30 or 90 seconds. Only GnRH neuron dendrons that displayed fast Ca^2+^ changes in response to KCl were subsequently tested with other compounds. Shifts in focal plane during the experiment were tolerated if they were less than 5%. To avoid substantial bleaching, imaging time was limited to 500 seconds around each puff application. To block action potential-dependent synaptic transmission, tetrodotoxin (TTX, 0.5-2 μM; Alomone Labs) was bath-applied during the whole of the experiment. Stock solutions of GABA (Sigma), muscimol (Tocris), RS-Baclofen (Sigma), and kisspeptin-10 (Calbiochem) were kept in −20 °C and diluted 1000 times from their stock solutions the day of the experiment.

### Data Collection and Analysis

Image acquisition was performed with Fluoview 1000 software. Frame scans (512 × 512 pixels) were performed on zoomed regions at a 0.9-Hz frame rate with the lowest possible laser power. Image analysis was performed with Fluoview1000 software and ImageJ. Regions of interest (ROIs) were drawn around GnRH neuron fibers and the percentage change in GCaMP6 fluorescence calculated as follows: GCaMP6 ΔF% = 100 × [(F − F0)/F0], where F is the average fluorescence of ROI in each consecutive frame and F0 is the average fluorescence of ROI in first 10 to 20 frames before test drug puffs. To quantify changes in fluorescence, a 5-second interval was used for the rapid increases observed with muscimol and GABA while a 20-second interval was used to quantify the longer-lasting suppressive actions. Tissue drift in the x-y axis was observed during imaging periods, and the drift was adjusted for by enlarging ROI or by Turboreg (ImageJ). Our prior studies with this preparation revealed that control applications of aCSF puffs can result in 4.5 ± 0.5% (mean ± SD) increases in [Ca^2+^] within dendrons (3). As such, we defined a threshold for a drug-induced change in [Ca^2+^] as requiring an increase or decrease in fluorescence greater than the aCSF control mean plus 2 SDs (ie, > 5.5%) (3).

All data are presented as mean ± SEM with “N” representing the number of mice and “n” showing the number of individual dendrons. Statistical analyses were performed with nonparametric paired-sample Wilcoxon signed ranks test or repeated-measures tests (Kruskal-Wallis or Friedman test with a post hoc Turkey test) or chi-square test. *P* < 0.05 was considered statistically significant.

## Results

### γ-Aminobutyric Acid Exerts Dynamic GABA_A_ and GABA_B_-mediated Effects on [Ca^2+^] in Gonadotropin-Releasing Hormone Neuron Dendrons Across the Estrous Cycle

**Figure 1.**
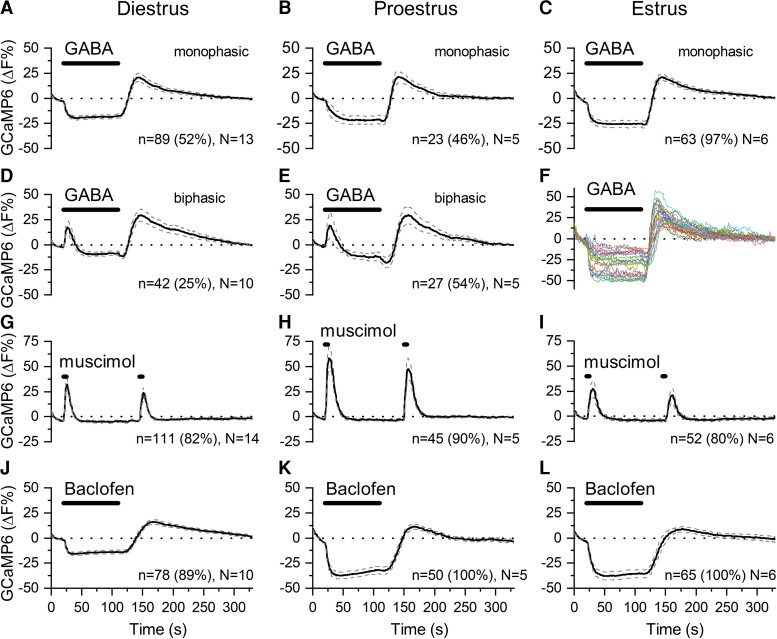
GABA regulates [Ca^2+^] in GnRH neuron distal dendrons throughout the estrous cycle. A to E, Average [Ca^2+^] levels in dendrons from *Gnrh1*-Cre::GCaMP6s mice showing the effects of 90-second puffs of 80 μM GABA at A, diestrus; B, proestrus; and C, estrus. A to C show monophasic effects with a suppression of [Ca^2+^] and rebound increase at the end of the GABA application. D and E show biphasic effects with an abrupt increase followed by a prolonged suppression and rebound increase. F shows color-coded traces from individual dendrons in an estrous brain slice showing monophasic responses. G to I, Average [Ca^2+^] levels in dendrons showing the abrupt stimulatory effects of 5-second puffs of 12.5 μM muscimol at G, diestrus; H, proestrus; and I, estrus. J to L, Average [Ca^2+^] levels in dendrons showing the prolonged suppressive effects of 90-second puffs of 20 μM baclofen at J, diestrus; K, proestrus; and L, estrus. Dotted lines indicate 95% CIs. Animal number is N. Number (and percentage) of dendrons responding in this manner represented by “n.”

**Figure 2.**
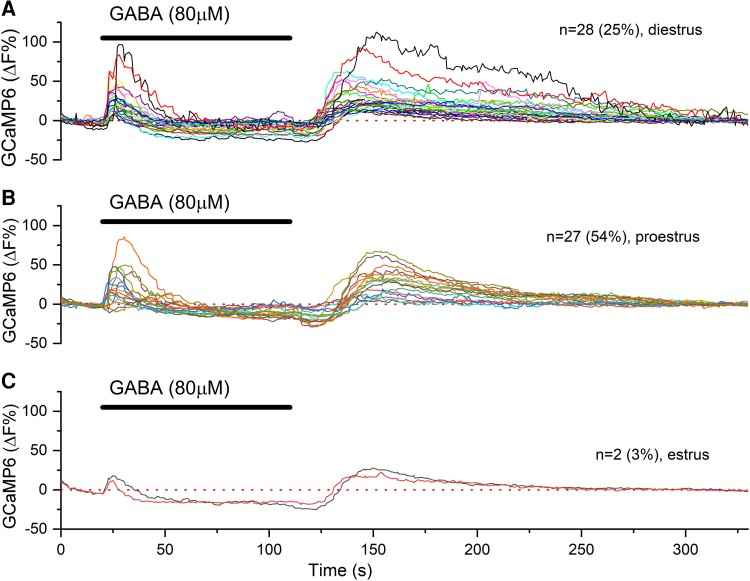
Examples of biphasic GABA responses in individual GnRH neuron distal dendrons. A to C, Color-coded traces from individual dendrons in brain slices from A, diestrous; B, proestrous; and C, estrous mice. Only 2 of 65 dendrons were found to exhibit biphasic responses in estrus. Number (and percentage) of dendrons responding in this manner represented by “n.”

The effects of GABA on dendron GCaMP6 fluorescence were examined in slices prepared from AAV-injected male and female diestrous-, proestrous-, and estrous-stage *Gnrh1-Cre* mice. In diestrous mice, 2 different responses to 90-second puffs of 80 μM GABA were observed. The most common response, observed in 52% of dendrons (89/170 dendrons, N = 13 mice), was a robust and sustained decrease in fluorescence that was followed, after termination of the GABA puff, by a rebound increase (Fig. 1A). Despite the rebound, which is seen in all recordings, this response is classified as being a “monophasic” as it consists of only a suppression in GCaMP6 fluorescence in the presence of GABA. The second response, observed in 25% (42/170, N = 13) of dendrons, was classified as being “biphasic” as it consisted of an immediate abrupt increase in fluorescence followed by a sustained suppression, and then the rebound (Figs. 1D and 2A). The magnitude of suppression occurring during a biphasic response was approximately 50% of that occurring during a monophasic response (Table 1). Approximately one-quarter of dendrons (23%) exhibited no response to GABA in diestrous mice.

**Table 1.** GABA-induced changes in calcium concentration of dendrons in female mice across the estrous cycle

		Biphasic		Monophasic
		GABA_A_	GABA_B_	GABA_B_
Diestrus	% Change GCaMP6 F	16.1 ± 3.0	−9.0 ± 1.0	−18.70 ± 0.94
	% Responding	25%*^a^*		52%
Proestrus	% Change GCaMP6 F	17.95 ± 3.93	−9.34 ± 0.25	−21.66 ± 1.97
	% Responding	54%*^a^*		46%
Estrus	% Change GCaMP6 F	12.64 ± 0.75	−13.41 ± 1.18	−25.55 ± 0.92
	% Responding	3%		97%

Mean ± SEM change in GCaMP6 fluorescence attributable to GABA_A_ or GABA_B_ responses and percentage of dendrons exhibiting monophasic or biphasic response to GABA in diestrus (n = 170, N = 13), proestrus (n = 50, N = 5) and estrus (n = 65, N = 6) mice.

Abbreviation: GABA, γ-aminobutyric acid.

a *P* < 0.001 compared to all other stages of estrous cycle (chi-square test; > 2 × 2 table).

To assess the contributions of GABA_A_ and GABA_B_ receptors to responses, dendrons were tested with 5-second puffs of muscimol (12.5 μM) and 90-second puffs of baclofen (20 μM). Muscimol evoked abrupt and transient increases in fluorescence in the majority (82%) of dendrons (111/135, N = 14) (Fig. 1G) with no delayed effects. In contrast, baclofen generated a marked and sustained suppression in GCaMP6 fluorescence in 89% of dendrons (78/88, N = 10) (Fig. 1J) followed by a rebound. These data indicate that the great majority of dendrons in diestrous mice express functional GABA_A_ and GABA_B_ receptors and that these receptors underlie the different multiphasic effects of GABA on dendron [Ca^2+^].

In proestrous mice, the same types of responses to GABA were observed with 23 of 50 dendrons (46%, N = 5) exhibiting a monophasic suppression (Fig. 1B) and 27 of 50 dendrons (54%, N = 5) having biphasic responses (Figs. 1E and 2B). When assessed with the selective receptor agonists, essentially all dendrons (45/50, N = 5) responded to GABA_A_ receptor activation with transient increases in fluorescence (Fig. 1H) and, similarly, 100% of dendrons (n = 50, N = 5) exhibited a marked suppression upon GABA_B_ receptor activation (Fig. 1K).

In estrous mice, nearly all dendrons (63/65, N = 6) exhibited the monophasic suppression in GCaMP6 fluorescence in response to GABA (Fig. 1C and 1F) with only 2 of 65 (3%) dendrons showing the biphasic response (Fig. 2C). In response to the agonists, 80% (52/65, N = 6) and 100% (n = 65) of dendrons in estrous mice showed typical transient stimulatory and sustained inhibitory responses to muscimol and baclofen, respectively (Fig. 1I and 1L).

The muscimol and baclofen data indicate that the vast majority (80%-100%) of dendrons express functional GABA_A_ and GABA_B_ receptors across the estrous cycle. Whereas the GABA_B_ response could be found alone (monophasic responses), the GABA_A_ response was only ever observed with a subsequent GABA_B_ response (biphasic). Marked fluctuations occurred in the efficacy of GABA activation at these 2 receptors across the cycle. In terms of the percentage of dendrons exhibiting transient increases, this fluctuated from 3% in estrus to 25% in diestrus and 54% in proestrus (*P* < 0.001, chi-square test; see Table 1). In contrast, the percentage of dendrons exhibiting GABA_B_ responses (either as monophasic or biphasic changes) was less variable with 77% of dendrons in diestrus and 100% during both proestrus and estrus (see Table 1).

**Figure 3.**
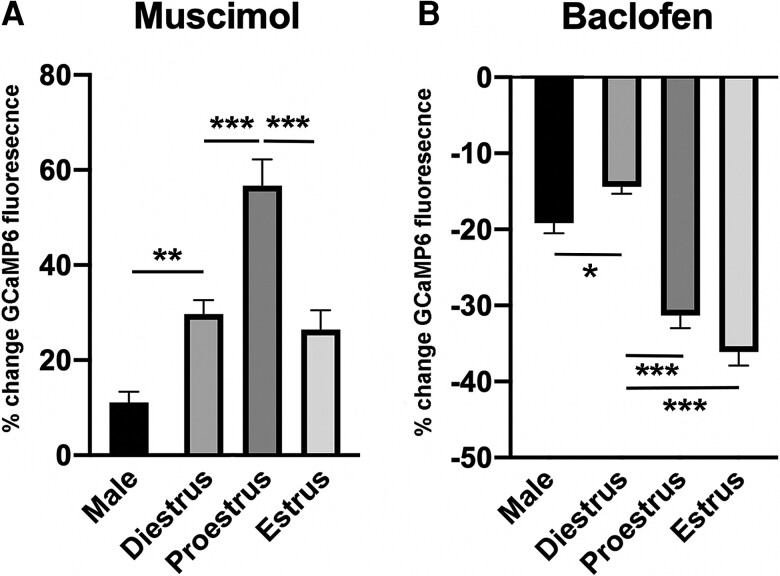
Estrous- and sex-dependent changes in dendron [Ca^2+^] responses to muscimol and baclofen. A, Histogram showing percentage increase in dendron [Ca^2+^] evoked by 12.5 μM muscimol. B, Histogram showing percentage decrease in dendron [Ca^2+^] evoked by 20 μM baclofen. *P < 0.05, **P < 0.01, ***P < 0.001 One-way analysis of variance with post hoc Tukey tests.

We also noted that the magnitude of the fluorescence response to muscimol and baclofen varied across the cycle. The dendron response to muscimol was increased by 1.9-fold in proestrous compared to diestrus and estrus (Fig. 3A; one-way analysis of variance [ANOVA], *F* = 17.3, *P* < 0.0001; post hoc Tukey test; *P* = 0.0003) while the suppression evoked by baclofen was approximately 2-fold greater in proestrus and estrus compared to diestrus (Fig. 3B, one-way ANOVA, *F* = 82.3, *P* < 0.0001; post hoc Tukey test *P* < 0.0001).

### Sex Differences Exist in γ-Aminobutyric Acid Regulation of the Dendron

**Figure 4.**
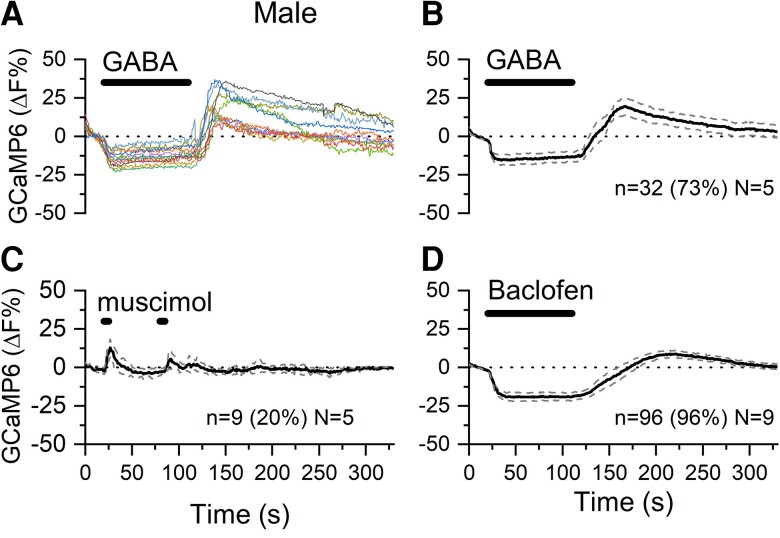
GABA regulates [Ca^2+^] in GnRH neuron distal dendrons of male mice. A, Color-coded traces from individual dendrons in brain slices from a male mouse exhibiting only monophasic suppressive responses. B to D, Average [Ca^2+^] levels of dendrons from male mice showing responses to B, 80 μM GABA; C, 12.5-μM muscimol; and D, 20 μM baclofen. Dotted lines indicate 95% CIs. Animal number is N. Number (and percentage) of dendrons responding in this manner represented by “n.”

In males, 90-second puffs of GABA generated the monophasic inhibitory response in 73% of dendrons (32 of 44, N = 5) with the remaining dendrons showing no response (Fig. 4A and 4B). Although this indicated the presence of only GABA_B_ receptors on male dendrons, we went ahead and assessed the effects of muscimol and baclofen. Transient, very small amplitude increases in fluorescence were observed in response to muscimol in 9 of 44 (20%) dendrons in 2 of the 5 male mice (Fig. 4C). In contrast, essentially all dendrons (96/100 tested, N = 9) responded to baclofen with the standard sustained suppression of fluorescence followed by a rebound increase (Fig. 4D).

In addition to a significantly reduced number of dendrons responding to muscimol in the male (20%) compared with females (82-90% in females; *P* < 0.001, chi-square test), male dendrons also exhibited a much-reduced muscimol-evoked increase in GCaMP6 fluorescence compared to females (Fig. 3A; one-way ANOVA, *F* = 17.3, *P* < 0.0001; Tukey tests *P* = 0.0092 (diestrus), *P* < 0.0001 (proestrus), *P* = 0.0907 (estrus). The overall percentage of dendrons exhibiting GABA_B_ responses was not different between the sexes (96% males, 89% diestrus females) and the magnitude of dendron GABA_B_ responses in males were intermediate between those of cycling females (see Fig. 3B).

### GABA_A_ Receptor Activation Generates Transient Desensitizing Actions in the Dendron

**Figure 5.**
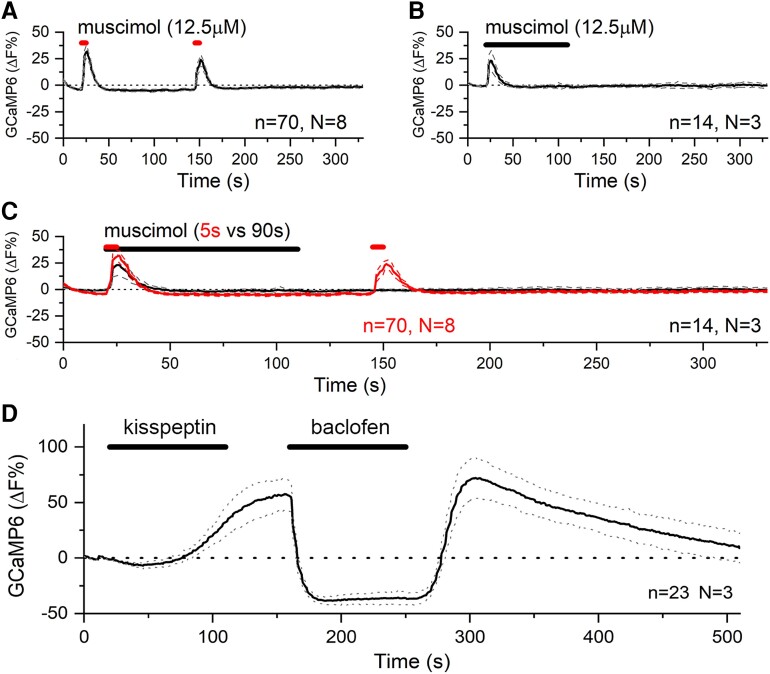
Muscimol generates transient stimulatory actions on [Ca^2+^] in GnRH neuron dendrons. A, Effects of repeated 5-second puffs of muscimol on dendron [Ca^2+^] in brain slices from diestrous mice (N = 8). B, Effects 90-second puffs of muscimol on dendron [Ca^2+^] in brain slices from diestrous mice (N = 3). C, Overlay of the average responses to 5-second (red) and 90-second (black) muscimol. D, Effect of 20 μM baclofen on kisspeptin (100 nM)-evoked [Ca^2+^] release (N = 3, 2 diestrus and 1 male mouse). Dotted lines indicate 95% CIs. Animal number is N and number of dendrons represented by “n.”

Responses to GABA in females reflect the net actions of GABA_A_ and GABA_B_ receptor activation on intracellular [Ca^2+^] in each dendron. It was unclear whether the transient nature of the stimulatory GABA response in biphasic responses resulted from possible desensitization of the GABA_A_ receptor or reflected the slower time dynamic for GABA_B_ receptor suppression of intracellular [Ca^2+^] to be effective. To test this, diestrous female dendrons exhibiting biphasic responses to GABA were identified and tested both with 5- and 90-second puffs of 12.5 μM muscimol. As expected, the 5-second puffs generated the normal repeatable, short transient increases in GCaMP6 fluorescence (Fig. 5A, n = 70, N = 8). However, when dendrons were exposed to 90-second duration muscimol, the exact same transient, stimulatory effects were observed despite the prolonged puff (Fig. 5B and 5C; n = 14, N = 3). This indicates that the transient nature of GABA_A_ receptor activation on the dendron is independent of GABA_B_ receptor activation.

### GABA_B_ Receptor Suppresses Kisspeptin Activation of the Dendron

Consistent with its role in pulse generation, puffs of kisspeptin evoke a potent activation of intracellular [Ca^2+^] in the dendron (3, 13). We examined whether the GABA_B_ receptor suppression described earlier would be sufficient to inhibit the stimulatory effects of kisspeptin on the dendron. Baclofen (20 μM) applied for 90 seconds 2 minutes after a 90-second kisspeptin (100 nM) puff was found to acutely and completely abolish the kisspeptin-evoked increase in intracellular [Ca^2+^] (Fig. 5D; n = 23 dendrons from 2 diestrous and 1 male mouse).

## Discussion

**Figure 6.**
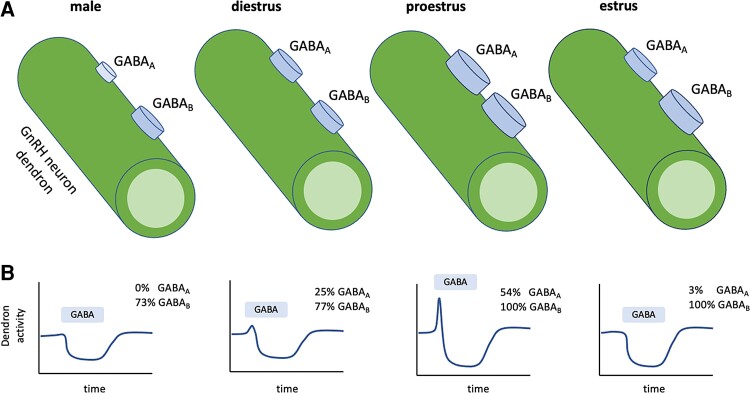
Schematic summary of GABA regulation of GnRH neuron dendron. A, The great majority (> 80%) of dendrons in female mice express functional GABA_A_ and GABA_B_ receptors but their efficacy in modulating [Ca^2+^] varies across the cycle. The size of the “receptor disk” indicates the relative effect of activating GABA_A_ and GABA_B_ receptors (from Fig. 3). For example, the magnitude of GABA_A_ and GABA_B_ responses are maximal on proestrus while GABA_A_ responses are minimal in males. B indicates the average profile of dendron [Ca^2+^] response to GABA release alongside the percentage of dendrons exhibiting the transient stimulatory (GABA_A_) and persistent inhibitory (GABA_B_) responses.

We report here that the activity of the GnRH neuron dendron is robustly modulated by GABAergic transmission. These actions are almost certainly direct on the dendron as they were recorded in the presence of TTX, and electron microscopic imaging has revealed the presence of symmetric, stereotypical GABAergic, synapses on the GnRH neuron distal dendron (4). The GABA_B_ receptor was the dominant GABA receptor subtype modulating the activity of the GnRH neuron distal dendron with baclofen evoking a marked suppression of intracellular calcium levels. More than three-quarters of dendrons exhibited functional GABA_B_ receptors both in males and females, with the proestrus and estrus stages of the cycle exhibiting the strongest inhibition. In contrast, the functional expression of GABA_A_ receptors was more limited with very little efficacy in males while effects in females were most prominent during proestrus with approximately 50% of dendrons responding. Interestingly, GABA_A_ receptor activation is stimulatory at the distal dendron, similar to that found for the proximal dendrites and cell bodies of GnRH neurons (15, 16). Together, these observations indicate that GABA inputs provide a robust, primarily GABA_B_-mediated inhibitory influence on dendron activity in males and in females throughout the estrous cycle (Fig. 6).

The intracellular calcium levels of most dendrons are robustly suppressed by GABA_B_ receptor activation. Ligand binding to the GABA_B_ receptor results in disassociation of the Gα_i/o_ subunit and the Gβγ dimer that, respectively, activate G protein–gated inwardly rectifying potassium (GIRK) channels and suppress voltage-gated calcium channels (VGCCs) (17). The channels and G proteins expressed by the dendron are not established, but it is possible that the GABA_B_ receptor uses both potassium and calcium channel modulation to achieve such a robust fall in intracellular calcium levels. The hyperpolarizing effect of GABA_B_ receptor activation at the GnRH neuron cell body is mediated primarily by GIRK channels (18, 19). It was interesting to observe that baclofen completely suppressed the kisspeptin-evoked increase in dendron intracellular [Ca^2+^] that is known to be dependent on VGCCs (13). All dendrons displaying the GABA_B_-mediated suppression showed a prompt rebound in intracellular calcium levels when the GABA or baclofen puff was stopped. This very likely represents a rebound in excitability mediated by hyperpolarization-activated cation and calcium channels known to be expressed by GnRH neurons (5).

Some dendrons exhibited rapid increases in intracellular calcium in response to GABA_A_ receptor activation. These effects were transient, lasting about 25 seconds, and independent of the duration of GABA_A_ receptor activation or accompanying GABA_B_ receptor activation. This is reminiscent of the GnRH neuron cell body and proximal dendrites that exhibit similar transient GABA_A_ receptor-mediated excitations following exogenous or endogenous GABA release (16, 20). Interestingly, the corticotropin-releasing hormone neuron axon terminals within the median eminence have also been reported to be depolarized by GABA_A_ receptor activation (21).

In the dendron, GABA stimulation was observed in the presence of TTX indicating that GABA_A_-mediated membrane depolarization was likely sufficient to activate low-threshold VGCC to abruptly increase calcium influx. In contrast to the GABA_B_ receptor, the GABA_A_ receptor exhibited marked sex-specific and estrous cycle–dependent contributions to dendron GABA responses. Stimulatory effects to GABA were observed in 3%, 25%, and 54% of dendrons in estrous, diestrous, and proestrous females, respectively, and never in males (see Fig. 6). The cycle-dependent changes in dendron responses are very likely attributable to plasticity in GABA receptors rather than more fundamental biophysical changes in the dendron as action potential activation of intracellular calcium in the dendron is equivalent across the estrous cycle (13). Although the GABA_A_ receptor subunits expressed by GnRH neurons have been well characterized (22–24), nothing is presently known about the trafficking of GABA_A_ receptor subunits to the dendron. Nevertheless, it is interesting to note that transcript levels of multiple GABA_A_ receptor subunits, as well as the GABAB-R1 subunit, are elevated in GnRH neuron somata on proestrus (24).

Dendrons never displayed a GABA_A_ response without a subsequent GABA_B_-mediated suppression, suggesting that GABA_A_ receptors are always colocalized with GABA_B_ receptors on the dendron. The same is not true for GABA_B_ receptors, which can often appear to be the sole functional GABA receptor expressed by a dendron. Interestingly, the concordance between the 2 different GABA responses and the presence of functional GABA_A_ and GABA_B_ receptors was quite variable. For example, in males, no transient stimulatory effects were seen in response to GABA and, in agreement, only 20% of dendrons exhibited very small stimulatory responses to muscimol. However, during estrous, muscimol evoked stimulatory responses from 80% of dendrons but GABA itself almost never generated a biphasic response. This suggests that, despite the presence of both GABA receptors, the GABA_B_ receptor is the dominant receptor determining the GABA response. There is a remarkable parallel between these findings and those found for the GnRH neuron proximal dendrites (20). In that case, the observed balance of GABA_A_ vs GABA_B_ response shifts with distance out along the dendrite in favor of the GABA_B_ receptor, again, despite the presence of both receptors along the entire dendrite. Thus, the strong predominance of functional GABA_B_-mediated inhibition at the level of the distal dendron may represent a gradual continuum from the proximal dendrite. One consequence of apparently “silent” GABA_A_ receptors on the distal dendron would be that of providing a current shunt so that the GABA_B_ receptor–mediated suppression is less robust. Indeed, we found that monophasic GABA suppressions in calcium were half the amplitude of the biphasic suppression.

The functional role of GABAergic modulation of the distal dendron in vivo is unknown. However, in concordance with the current observations, early studies examining GnRH release from “arcuate nucleus–median eminence” fragments reported that GABA_A_ receptor activation stimulated GnRH secretion whereas GABA_B_ receptor activation inhibited release (25). One role for the dominant inhibitory GABA_B_ effects on the distal dendron may be in counterbalancing or modulating the excitatory drive provided by the kisspeptin pulse generator (3, 13). We show here that GABA_B_ receptor activation completely and acutely abolishes kisspeptin activation of the dendron. Interestingly, mice with a global knockout of the GABA_B_ receptor exhibit increased GnRH pulse frequency and, in response to a constant kisspeptin stimulus, GnRH pulses have an increased mass per pulse in these mice (10, 26). Thus, it is possible that GABAergic inputs to the distal dendron provide a mechanism for the regulation of pulse generation at the level of the dendron itself. For example, the way in which corticotropin-releasing hormone neurons suppress pulsatile LH secretion remains unknown and does not appear to involve direct regulation of the arcuate kisspeptin neurons (27). However, the locations of GABAergic cell bodies innervating the GnRH neuron distal dendron are unknown and the delineation of functional roles for GABAergic inputs to the dendron in vivo will be challenging technically.

As the distal dendron is responsible for conveying action potentials to the median eminence, it is possible that GABAergic drive to this compartment may regulate the timing or amplitude of the GnRH surge. This is the one time during the estrous cycle where significant GABA_A_-mediated stimulatory effects are seen at the dendron, so GABAergic drive may facilitate release. In this case, however, the GABA drive would have to occur as short, phasic bursts of GABA release to avoid invoking the GABA_B_-mediated inhibition. In this respect, the distal dendron could operate as a bidirectional input filter with phasic GABA release promoting, and tonic GABA release suppressing, GnRH release. It will be important in future studies to discern the functional significance of afferent GABAergic input to the distal dendron and roles of GABA_B_ receptors more generally in GnRH neurons. This is likely to require selective GnRH neuron and GnRH neuron compartment modulation strategies.

In summary, we report here a new location for the GABAergic modulation of GnRH secretion. The particularly robust suppression of distal dendron activity by GABA_B_ receptor activation provides a novel kisspeptin-independent pathway through which both pulsatile and surge modes of GnRH secretion may be modulated to regulate fertility.

## Data Availability

Some or all data sets generated during and/or analyzed during the present study are not publicly available but are available from the corresponding author on reasonable request.

## Abbreviations

AAV: adeno-associated virus
aCSF: artificial cerebrospinal fluid
ANOVA: analysis of variance
GABA: γ-aminobutyric acid
GIRK: G protein–gated inwardly rectifying potassium
GnRH: gonadotropin-releasing hormone
LH: luteinizing hormone
ROI: region of interest
s.c.: subcutaneously
TTX: tetrodotoxin
VGCC: voltage-gated calcium channel
